# Metabolome analyses of the rumen, feces and blood reveal separate and simultaneous impacts of increased dietary concentrate and hindgut starch flow in Holstein cows

**DOI:** 10.1007/s11306-026-02519-0

**Published:** 2026-10-03

**Authors:** Ezequias Castillo-Lopez, Markus Aigensberger, Rana Muhammad Atif, Patrick Biber, Thomas Hartinger, Sabina Raykova, Alejandra Guerrero-Lavin, Nicole Reisinger, Heidi E. Schwartz-Zimmermann, Franz Berthiller, Qendrim Zebeli

**Affiliations:** 1 Centre for Animal Nutrition and Welfare, University of Veterinary Medicine, 1210 Vienna, Austria; 2 Christian Doppler Laboratory for Innovative Gut Health Concept of Livestock, 1210 Vienna, Austria; 3 Department of Agricultural Sciences, BOKU University, 3430 Tulln, Austria; 4 Universidad Nacional Autonoma de Mexico, FES Cuautitlan, 54714 San Mateo Ixtacalco, Mexico; 5 dsm-firmenich, Animal Nutrition & Health R&D Center Tulln, 3430 Tulln an der Donau, Austria; 6 Present Address: Institute of Animal Science, University of Bonn, 53115 Bonn, Germany

**Keywords:** Cow, Blood, Feces, Rumen, Metabolome, Starch

## Abstract

**Introduction:**

The combined effects of increased dietary concentrate and hindgut starch supply on the cow metabolome remain poorly understood.

**Objective:**

To evaluate the impacts of high concentrate feeding and the impacts of increased hindgut starch supply on the metabolome of rumen, feces and blood of Holstein cows.

**Methods:**

Either a 40% (Control) or a 65% concentrate diet (High) was fed. In addition, cows were abomasally infused with either water or starch (3 kg/day). Metabolomic analysis of rumen, feces and blood were performed with HPLC-MS/MS and anion exchange chromatography coupled to high-resolution mass spectrometry.

**Results:**

Ruminal pH duration < 5.8 was 69 and 249 min/day for Control and High, respectively; fecal pH was 6.58 and 6.33 for water- or starch-infused cows, respectively. Independent of abomasal starch infusion, High increased ruminal concentration of several amino acids, nucleosides, and lipid-related metabolites, and enriched metabolic pathways for purine metabolism, urea cycle and amino acid metabolism. In feces, both High and abomasal starch infusion increased metabolites related to fatty acid and nitrogen metabolism, and enriched pathways for bile acid biosynthesis, amino acid metabolism, α-linolenic acid and biotin metabolism. In blood, Control enriched metabolic pathways for amino sugar metabolism, aspartate metabolism, fatty acid biosynthesis and malate-aspartate shuttle, regardless of abomasal starch infusion.

**Conclusion:**

High enhanced metabolic pathways mainly for ruminal nitrogen metabolism. Both High and abomasal starch infusion influenced fecal metabolic pathways; whereby, the increased ethanolamine suggests its potential as a biomarker for gut acidosis. Compared to abomasal starch infusion, High had greater impacts on the blood metabolome.

**Supplementary Information:**

The online version contains supplementary material available at https://doi.org/10.1007/s11306-026-02519-0.

## Introduction

In cattle fed high concentrate rations, production performance is enhanced thanks to greater supply of dietary fat, protein, and particularly starch (Della Rosa et al., 2025; Thoefner et al., 2004). While this is an expected outcome, complications associated to rumen and systemic animal health are also commonly observed, and have been difficult to eradicate (Plaizier et al., 2018; Coon et al., 2019). For example, the readily available carbohydrates increase rumen fermentation and acid production, leading to development of subclinical ruminal acidosis (SRA). In dairy cattle, the estimated rate of SRA prevalence in commercial herds ranges from 10 to 26% (Golder & Lean, 2024); thus, this disorder may impact not only individual cow health, but also overall herd profitability. Additionally, high concentrate feeding may lead to hindgut acidosis (HGA) because of increased fermentation in the lower gut (Abeyta et al. 2023a). This condition results from increased digesta passage and greater escape of starch from the rumen, which reaches the lower gut, where it is further fermented, lowering digesta pH (Abeyta et al. 2023b). Therefore, both SRA and HGA may contribute to detrimental effects on gut function, animal health and welfare, and potentially impair production performance.

Metabolomics studies have recently improved our understanding on the effects of high concentrate feeding on the rumen and systemic health of cattle by simultaneously addressing a large array of phenotypic outcome (Zhang et al., 2022; Zhao et al., 2025). For example, Saleem et al. (2012) demonstrated that high-grain diets (> 30%) result in increased rumen fluid concentrations of several toxic, and inflammatory compounds. More recent reports have shown that high concentrate feeding may also increase the levels of nucleotides and nitrogen compounds, and lead to enrichment of metabolic pathways responsible for nitrogen and amino acid metabolism in the ruminal metabolome (Castillo-Lopez et al. 2025b), which may be due to the greater energy and nitrogen supply in cows consuming the high concentrate rations. However, the concurrent effects of high concentrate feeding and increased hindgut starch flow on the metabolome are lacking in the scientific literature.

The fecal metabolite pattern has also been shown to be affected by high concentrate rations (Christodoulopoulos, 2025). Additionally, we have recently reported that cows with greater degree of SRA show increased presence of metabolites associated with fat, amino acids and starch metabolism in the feces, resulting in enrichment of metabolomic pathways for amino acid degradation and metabolism (Castillo-Lopez et al. 2025a). This alteration may reflect increased passage of undegraded feed to the lower gut, shifting the fecal microbial activity, leading to an altered end-product profile. Considering that HGA exacerbates the acidity in the hindgut, greater impacts of both high concentrate feeding and increased hindgut starch flow on the fecal metabolome would be expected, but knowledge on this aspect is currently limited.

In the blood, cattle fed high concentrate diets show evident perturbations in the content of several amino acids including phenylalanine, lysine, leucine, arginine, and valine (Saleem et al., 2012). Moreover, cattle experiencing SRA show greater levels of cholesterol esters, phosphatidylcholines, and enrichment of metabolic pathways for steroid and bile acid biosynthesis (Castillo-Lopez et al. 2025b). This is partly because of the greater energy supply and the change in fermentation in the rumen, which in turn influence the nutrients absorbed by the animal (Yost et al., 1977; Russell, 1998). In addition, changes in blood metabolic composition due to inflammatory response may be expected (Maeda et al., 2019). Overall, while the effects of SRA on blood composition have been explored, there is limited information on how blood metabolome is affected by HGA. Additionally, it is important to investigate whether simultaneous high concentrate feeding and greater hindgut starch supply lead to concomitant impacts on blood profile and metabolic pathways.

Preliminary results from this project reported in a companion paper showed that high concentrate feeding and hindgut starch supply have separate and interactive impacts on gut fermentation and microbiome (Biber et al., 2026). For example, those results revealed that the high concentrate diet shifted ruminal VFA profile, while decreasing pH, shifting the microbial community profile, lowering microbial diversity. Furthermore, results in the companion paper showed that the abomasal starch infusion decreased fecal pH, increased fermentation, and shifted the fecal microbiome. Specifically, the abomasal starch infusion increased the abundance of butyrate producers such as Blautia (OTU 508) and Lachnospiraceae (OTU 18). Additionally, results revealed that high dietary concentrate in combination with abomasal starch infusion had additive impacts, aggravating the alteration of the fecal microbiome by reducing microbial α-diversity. Thus, it is reasonable to infer that such impacts could lead to important changes in the gut and blood metabolome of the animals. Therefore, the aim of this study was to evaluate the separate and concomitant impacts of high concentrate feeding and increased hindgut starch supply on the metabolome and the underlying metabolic pathways of the rumen fluid, feces, and blood of Holstein cows. We hypothesized finding separate as well as combined detrimental impacts of high concentrate feeding and increased hindgut starch supply on the metabolome; specifically, high concentrate feeding and increased hindgut starch supply will result in larger number of metabolites associated with utilization of starch and protein as well as metabolites associated with systemic health complication.

## Materials and methods

This study was part of a larger experiment; results on ruminal and fecal fermentation as well as the gut microbiome are reported in a companion manuscript (Biber et al., 2026). The experiment included 9 Holstein cows fitted with ruminal cannula (#1C4” and #1C5” with rolled inner flange, Bar Diamond Inc., Parma, Idaho, USA; 3rd lactation, 724 ± 79 kg BW at the start of the experiment, and 198 ± 74 days in milk (DIM). Before the start of each experimental period, cows were adapted to their individual feeders and were fed a lactation diet containing 40% concentrate. The treatment groups evaluated were: (1) 40% concentrate lactation diet (Control), (2) Control with abomasal infusion of starch to induce HGA (Control + HGA), (3) 65% concentrate diet (High), and (4) High with abomasal infusion of starch (High + HGA). Cows were allocated to each treatment so that there is a balance in days in milk across treatments; in addition, the body weight of cows was taken into account so that BW among treatments is as balanced as possible.

The experiment was performed as a changeover experimental design with 2 experimental periods of 3 weeks each (Supplementary material Fig. 1). In the first week of each period, all 9 cows were fed the control diet; during this week, data and sample collections were conducted for Control. In the second week, 6 of the 9 cows were switched to the High diet. In the third week, the 3 cows receiving Control as well as 3 of the 6 cows receiving High were infused daily with 3 kg of starch into the abomasum to induce HGA. Thus, in the third week, data and sample collection was conducted for treatments of Control + HGA, High, and High + HGA. Between the 2 experimental periods, there was a 3-week washout interval where cows consumed the control diet. In the second experimental period, the treatments were switched among cows, and measurements were performed again.

### Animal housing and feeding

Cows were kept in a free-stall barn, which allowed free movement and normal herding behaviour. The stall was equipped with 15 deep litter cubicles (2.6 × 1.25 m, straw litter). The cows had *ad libitum* access to water and a salt block during the entire experiment (except during sampling times). The feed bins were randomly allocated to the cows before the start of the experiment and each cow had access to only one feed bin throughout the experiment. All cows were trained to access the allocated feed bin by using an ear tag transponder. Each feed bin was equipped with an electronic scale (Insentec, B. V. Marknesse, Netherlands). Thus, individual feed intake was recorded throughout the experiment.

The rations were prepared daily in the morning using an automated feeding system (Trioliet Triometrci T 15, Oldenzaal, the Netherlands) and offered to the cows in their individual feed bins, which were refilled in the afternoon. Because of low moisture content in the feed ingredients, a calculated amount of water was added to the diet during mixing, targeting a DM content of total mixed ration (TMR) of 45%. The DM content of silages and TMR were determined weekly by drying samples at 103 °C for 24 h to ensure appropriate inclusion of water in the diet and fulfill the desired DM content. The concentrate mixture of diets contained highly degradable grains such as wheat/triticale, corn grain, and was formulated to meet the cows’ requirements for energy and all nutrients (GfE, 2001).

Cow body weights were recorded at the initiation and at end of the study. These measurements were performed following the morning milking and prior to the morning first feeding, as cows returned from the milking parlour to the research area.

### Collection of feed samples and analysis of chemical composition

Fresh feed samples were collected on a weekly basis. Forages and TMR were immediately stored at −20 °C, while concentrate ingredients were stored at room temperature for later analysis for chemical composition. All nutrient analysis of feed samples were conducted in duplicate according to the German Handbook of Agricultural Experimental and Analytical Methods (VDLUFA, 2012). The DM of wet feed samples was measured by forced-air drying at 65 °C for 48 h. Afterwards, the samples were ground through a 0.5 mm sieve (Ultra Centrifugal Mill ZM 200, Retsch, Haan, Germany) for proximate nutrient analysis. Residual moisture was further assessed by drying at 103 °C for 4 h (VDLUFA method 3.1). Ash content was measured through combustion in a muffle furnace at 580 °C overnight (method 8.1). Crude protein was determined using the Kjeldhal method, and ether extract was analyzed using the Soxhlet extraction system (Extraction System B-811, Flail, Switzerland). Neutral detergent fiber and acid detergent fiber contents were measured using VDLUFA methods 6.5.1 and 6.5.2, respectively, with sodium sulphite treatment and expressed exclusive of residual ash. The fiber analysis included the use of heat-stable α-amylase, and both fiber fractions were measured using the Fiber Therm FT 12 (Gerhardt GmbH Co. KG, Königswinter, Germany). The starch content was determined following the manufacturer’s protocol using an enzyme-based total starch assay (K-TSTA 100 A kit, megazyme Ltd., Wicklow, Ireland) and a UV-180 Spectrophotometer (Shimadzu, Kyoto, Japan). The analyzed chemical composition of the diets is listed in Table 1.

**Table 1:** Ingredients and chemical composition for the normal lactation diet (Control) and the high concentrate diet (High) fed during the experiment (values stated as % of dry matter, unless stated otherwise)

		Diet				
Item		Control			High	
Ingredients						
Grass silage		30			15	
Corn silage		30			20	
Protein supplement^1^		19			17	
Energy and mineral supplement^2^		21			26	
Dairy supplement^3^		0			22	
TMR chemical composition (mean ± standard deviation)		*n* = 6			*n* = 4	
Dry matter, % as fed		35.6 ± 0.8			38.9 ± 1.1	
Crude protein, %		19.4 ± 0.4			19.7 ± 0.1	
Neutral detergent fiber, %		35.1 ± 0.4			30.2 ± 0.4	
Acid detergent fiber, %		22.9 ± 0.3			19.1 ± 0.6	
Starch, %		20.7 ± 1.1			26.9 ± 0.5	
Ether extract, %		2.8 ± 0.1			3.1 ± 0.1	
Ash, %		8.1 ± 0.1			7.4 ± 0.3	
Particle fraction, % retained						
Long		24.2 ± 0.8			10.2 ± 2.7	
Medium		42.1 ± 2.3			40.2 ± 3.5	
Short		23.1 ± 3.9			35.1 ± 2.7	
Fine		10.6 ± 5.4			14.5 ± 8.9	
pef^4^ > 8 mm		0.66 ± 0.01			0.50 ± 0.1	
peNDF^5^ > 8 mm		23.2 ± 0.1			15.1 ± 0.2	

^1^Rindastar 39% XP Schaumann GmbH contained rapeseed meal, dried distillers’ grains with solubles, heat-extracted soybean meal, urea, and molasses. Chemical composition per kg: crude protein (40.0%), fat (3.6%), crude fiber (9.5%), ash (8.0%), net energy of lactation (6.7 MJ), calcium (0.9%), phosphorus (1.0%), sodium (0.25%), and magnesium (0.5%), vitamin A (9000 IU), vitamin D3 (1800 IU), vitamin E (30 mg), iodine (calcium iodate, anhydrous) 2.9 mg, cobalt (coated cobalt (II)carbonate granules) 0.4 mg, copper (copper (II)sulfate, pentahydrate) 10 mg.

^2^Rindastar SM Vet Schaumann GmbH contained maize, barley, wheat, calcium carbonate, sodium chloride, magnesium oxide, monocalcium phosphate, sodium bicarbonate, a premix with vitamins, and trace elements. Chemical composition per kg: crude protein (9.0%), fat (3.0%), crude fiber (2.8%), ash (9.5%), net energy of lactation (6.9 MJ), calcium (1.8%), phosphorus (0.36%), sodium (0.75%), magnesium (0.47%), vitamin A (45000 IU), vitamin D3 (9000 IU), vitamin E (150 mg), iodine (calcium iodate, anhydrous) 15 mg, cobalt (coated cobalt(II)carbonate granules) 2.0 mg, copper (copper (II)sulfate pentahydrate) 90 mg, copper (copper (II)glycine chelate hydrate) 30 mg, manganese (manganese(II)oxide) 164 mg, manganese (glycine manganese chelate, hydrate) 44 mg, zinc (zinc oxide) 250 mg, zinc (glycine zinc chelate, hydrate) 90 mg, selenium (sodium selenite) 2.4 mg.

^3^Kuhkorn Kompakt 19 Garant GmbH contained maize, rapeseed meal, wheat, dry distillers’ grains, wheat bran, maize gluten feed, barley, sugar beet molasses, calcium carbonate, sodium chloride, und magnesium oxide. Chemical composition per kg: crude protein (19.0%), fat (3.5%), crude fiber (6.0%), ash (6.0%), net energy of lactation (7.0 MJ), calcium (0.8%), phosphorus (0.65%), sodium (0.2%), magnesium (0.3%), vitamin A (6000 IU), vitamin D3 (900 IU), vitamin E (20 mg), copper (copper (II)sulfate pentahydrate) 11 mg, zinc (zinc sulfate, monohydrate) 15 mg, zinc (zinc oxide) 38 mg, manganese (manganese (II)oxide) 30 mg, iodine (calcium iodate, anhydrous) 1.2 mg, cobalt (coated cobalt (II)carbonate granules) 0.5 mg, selenium (sodium selenite) 0.3 g.

^4^Physical effectiveness factor.

^5^Physically effective neutral detergent fiber.

The particle size distribution of the diets was analyzed following the method of previous researchers (Kononoff et al., 2003), using a modified Penn State Particle Separator with three sieves (19.0 mm, 8.0 mm, and 1.18 mm) and a solid bottom pan. Physically effective fiber (peNDF) and the physically effective factor (pef) were determined based on the approach reported by other investigators (Beauchemin et al., 2003). In brief, peNDF was calculated by multiplying the diet’s NDF content by its corresponding pef. The pef (ranging from 0 to 1) was defined as the sum of the proportions of particles retained on a specific sieve: particles on the 19.0 mm and 8.0 mm sieves were used to calculate pef > 8 mm, and particles on the 19.0 mm, 8.0 mm, and 1.18 mm sieves were used for pef > 1.18 mm.

### Measurement of ruminal pH

Details on the measurements of ruminal and hindgut fermentation and pH have been reported in the companion manuscript (Biber et al., 2026). Briefly, ruminal pH was measured throughout the experiment every 15 min in each cow with the Lethbridge Research Centre Ruminal pH Measurement System (LRCpH; Dascor Inc., CA, USA; Penner et al., 2006). Calculations, as well as conversion of measured millivolts to pH, were performed in excel following a similar method used by Castillo-Lopez et al. (2014a, 2014b). Calibrations of the rumen pH systems were performed before inserting them into the rumen and after weekly download of pH data. The calibration values were used to correct ruminal pH drift. Regarding the definition of SRA in the present study, SRA occurrence was identified as the significant reduction of ruminal pH using the variables of time duration with pH below the threshold 5.8 in a 24 h interval as well as the averaged mean ruminal pH in a 24 h interval measured with the indwelling systems.

### Abomasal starch infusions

To induce hindgut acidification, the corresponding cows were abomasally infused with starch via ruminal cannula throughout week 3, following an approach used by Spires et al. (1975) and Gressley et al. (2006). Briefly, daily starch infusions of 3 kg of starch (1:1 corn to wheat starch weight ratio) were performed three times a day (1 kg each time dissolved in 4 L of water) at 0, 6 and 12 h relative to the first morning feeding. Cows not receiving starch were infused only the same volume of water. In the present study, the abomasal infusion lines could be inserted without removing the rumen contents. Therefore, there was no risk of excessive exposure of the ruminal contents to air. We defined HGA occurrence as the significant reduction of mean fecal pH measured at timepoints 0, 6, and 12 h relative to the first morning meal. In general, the reduction in fecal pH was similar to values reported by other researchers in conditions characterized as hindgut acidification (Plaizier et al., 2022; Sanz-Fernandez et al., 2024).

### Collection of ruminal fluid

Ruminal fluid samples were collected at the end of week 1 and week 3 of each experimental period (same collection days for feces and blood samples). These samplings were conducted in the morning before the first meal. To do so, the whole rumen contents were collected from 4 different regions of the rumen (caudal ventral sac, cranial ventral sac, and 2 samples from the feed mat in the dorsal rumen) and composited to obtain a representative sample per cow, per sampling timepoint per treatment (Castillo-Lopez et al., 2014a, 2014b; Ramirez Ramirez et al., 2015). The rumen contents were then strained through 4 layers of gauze; approximately 1.8 mL of strained rumen fluid were pipetted in a 2-mL cryotube, and immediately snap frozen in liquid nitrogen; at the end of the samplings, these vials were frozen at − 80 °C for later metabolomics analyses.

### Collection of fecal samples

Fecal samplings were performed by collecting grab samples rectally. The sample collections were conducted in the morning before feeding. Around 2 g of the collected feces were placed in cryotubes using a spatula previously sterilized with 70% ethanol, and they were immediately snap frozen in liquid nitrogen. Collected samples were then stored at − 80 °C until further analysis for metabolomic composition.

### Collection of blood samples

Blood samples were collected from the jugular vein. These samplings were conducted in the morning before the first meal was offered. After sample collections, the filled serum vacutainers (9 mL, Vacuette, Greiner Bio-One, Kremsmuenster, Austria) were stored at room temperature for approximately 1.5 h. Then, all vacutainer tubes were centrifuged at 2,000 × g for 15 min at 4 °C (Centrifuge 5804 R, Eppendorf; Hamburg, Germany), and the supernatant was pipetted into 2 mL cryotubes (Eppendorf). Then, these samples were placed on ice during pipetting, and stored at − 80 °C. Analyses for blood metabolomic profile were conducted after completion of the experiment.

### Metabolomic analyses of rumen fluid, fecal and blood serum samples

Metabolomics analyses were performed as described by Duszka et al. (2026). Briefly, the respective matrices were processed as follows. For work-up of fecal samples, 100 mg of feces was extracted with 1.6 mL of acetonitrile (ACN)/water (80:20, *v*:*v*) by vortexing for 30 s, shaking at 180 rpm at 4 °C for 25 min, sonication in ice water at 70 W for 5 min, and centrifugation at 14,350 × g for 10 min. The resulting supernatant was measured undiluted and additionally after 1:10 (*v*:*v*) dilution by reversed phase liquid chromatography (RP-HPLC) and hydrophilic interaction chromatography (HILIC) coupled to tandem mass spectrometry (MS/MS) as originally described by Xu et al. (2025).

For RP-HPLC-MS/MS and HILIC-MS/MS analysis of serum and rumen fluid samples, 200 µL of sample was mixed with 800 µL of extraction solvent (ACN for rumen fluid, isopropanol for serum) and vortexed for 30 s. Samples were centrifuged at 12,500 rpm for 10 min at 4 °C, and the supernatant was diluted 1:5 and 1:100 (*v*:*v*) for subsequent analysis. Both dilutions for each matrix were analyzed by both RP-HPLC and HILIC-MS/MS methods according to Xu et al. (2025). Carboxylic acids, sugar related compounds and nucleotides in serum samples were analyzed by anion exchange chromatography coupled to high-resolution mass spectrometry (AIC-HR-MS). To this end, 20 µL of serum was spiked with 10 µL of internal standard solution (5 mg/L of fully ^13^C-labelled acetic acid, propionic acid and butyric acid), 470 µL of ACN/water (80:20, *v*:*v*) was added and analytes were extracted by shaking at 4 °C for 10 min and centrifugation at 14,350× g for 10 min.

In short, HPLC-MS/MS analyses were carried out on an Agilent 1290 series UHPLC system (Agilent, Waldbronn, Germany) coupled to a 6500 + QTrap mass spectrometer equipped with an IonDrive Turbo V^®^ source (Sciex, Foster City, CA, USA). The RP chromatography was performed on a Kinetex C18 column (150 × 2.1 mm, 2.6 μm, Phenomenex, Aschaffenburg, Germany) in gradient elution mode. The HILIC separations were carried out on a Kinetex HILIC column (150 × 2.1 mm, 2.6 μm, Phenomenex), also with gradient elution. Mass spectrometric detection was done in scheduled selected reaction monitoring mode with fast polarity switching after electrospray ionization. AIC-HR-MS analysis was carried out on a Dionex Integrion HPIC system coupled to a Q Exactive Orbitrap mass spectrometer (both Thermo Scientific, Waltham, MA, USA) as detailed by Ricci et al. (2022, 2024).

Analytes were quantified on the basis of calibration functions measured at the beginning and at the end of each measurement sequence, along with regular injections of a pooled quality control (QC) sample and a single calibration standard throughout the run. Drift correction was performed using loess regression on the QC sample or standard and peak areas were normalized accordingly. Subsequently, quantification was performed using linear or quadratic regression models based on manual revision. All steps were carried out with the QuantyFey application (Aigensberger et al., 2025). Metabolome output data can be found at https://doi.org/10.17632/2w4fk6vc3k.1.

### Statistical analyses

Statistical power analysis was performed following a similar approach reported by Kononoff and Hanford (2006). The results of statistical power analysis indicated a power ranging from 78 to 95% with an average of 85%; therefore, indicating sufficient statistical power to detect a difference when comparing the treatment groups.

Preliminary check and curation of metabolome output data included evaluations of peak areas and concentration of metabolites, and the presence of potential confounding molecules or contaminants. Using information generated from non-template vials (blanks), the blank-to-sample ratio was calculated to identify and remove background noise by dividing the average intensity of each blank by the intensity of the respective sample. A high ratio, suggesting domination by background noise, was excluded from further analysis. In accordance with Schiffman et al. (2019), a cut off sample-to-blank ratio of 4 was used to filter low-quality data.

The metabolome data of ruminal fluid, blood serum and fecal samples were statistically analyzed separately using MetaboAnalyst (v. 6.0), following data conversion, normalization and filtering steps as detailed previously (Pang et al., 2022). Principal coordinate analysis plots were generated to obtain an overall knowledge of the similarities among the metabolomic profiles of treaments. In doing so, for hypothesis testing, permutational multivariate analysis of variance (PERMANOVA) was also performed. Additionally, the statistical analyses evaluated the fixed effects of diet and abomasal infusion as well as their interaction. Therefore, the statistical model included the fixed effects of diet and abomasal infusion as well as the random effect of cow within treatment, which was considered as the experimental unit. The *P*-values were adjusted with the Benjamini-Hochberg method for false discovery rate. Statistical significance was declared when *P* ≤ 0.05 and tendency is indicated if *P* > 0.05 and ≤ 0.10.

Subsequently, based on the detected differential abundance of metabolites among treatments being compared within each sample type (for example, comparing Control vs. High on the blood metabolome), the analysis for prediction of metabolomic pathway enrichment was also performed using MetaboAnalyst (v. 6.0) and the dot plots indicating enrichment ratios were constructed. For this analysis, the *P*-values were adjusted with the Benjamini-Hochberg method for false discovery rate. Additionally, statistical correlation analyses were peformed between each treatment and the metabolites detected. The significance in correlation results was declared when *P* ≤ 0.05 and a tendency was indicated if *P* > 0.05 and ≤ 0.10.

## Results

### Ruminal and hindgut pH due to high concentrate diet and abomasal starch infusion

The results of pH measurements confirmed significant rumen acidification with the high concentrate diet (*P* < 0.05), which was reflected in greater time with ruminal pH < 5.8 (*P* < 0.05), with average duration of 69 and 249 min/d for Control and High, respectively. Additionally, mean ruminal pH was 6.26 and 6.09 for Control and High, respectively. These shifts were accompanied by greater proportions of propionate and lower acetate in High (*P* < 0.05). On the other hand, the abomasal infusion of starch resulted in lower fecal pH compared to cows without abomasal starch infusion, with average fecal pH of 6.58 and 6.33, for non-infused and infused cows, respectively (Supplementary material Fig. 2), which was accompanied by greater proportions of propionate and lactate (*P* < 0.05). Additionally, feeding the high concentrate diet tended (*P* = 0.07) to increase feed intake, with averages of 23.3, 22.8, 26.4 and 26.4 kg for Control, Control + HGA, High and High + HGA, respectively.

### Effect of high concentrate diet and abomasal starch infusion on the ruminal fluid metabolome

**Fig. 1:**
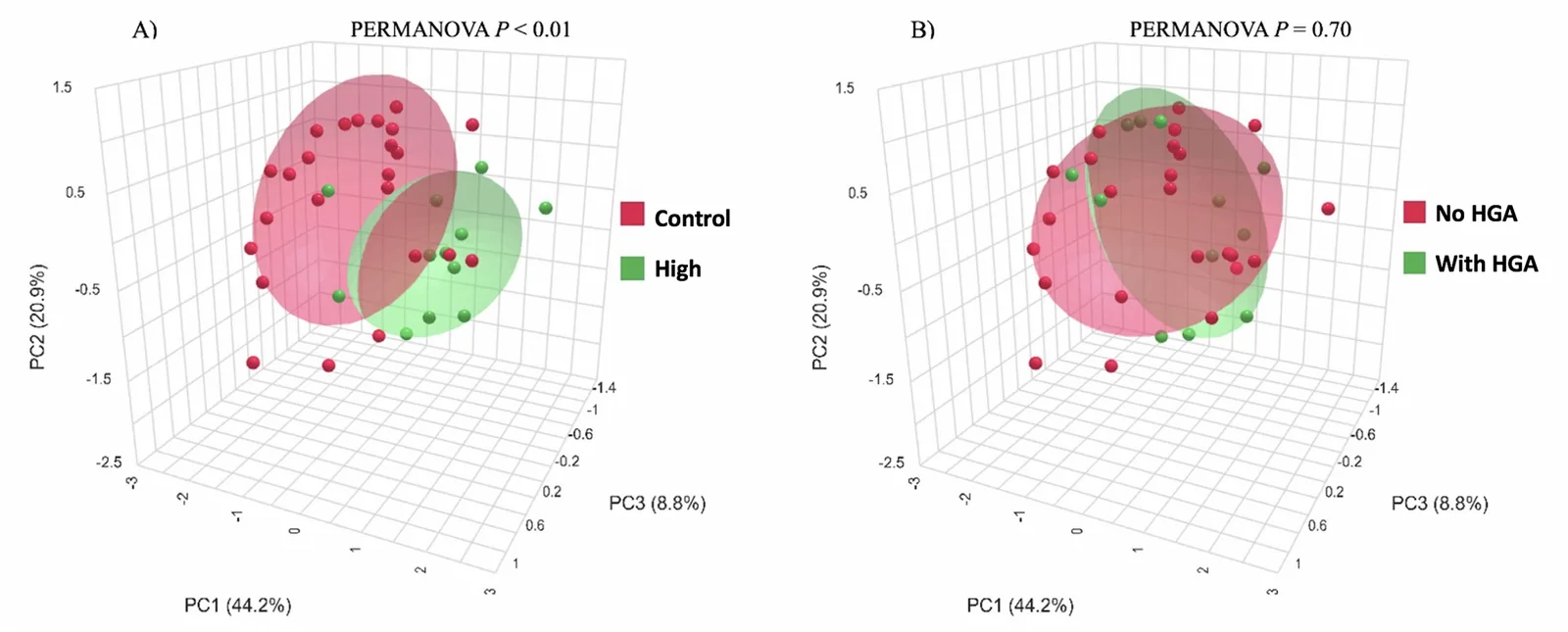
**A** Principal component plot of ruminal fluid metabolome comparing cows consuming a control diet with 40% concentrate (Control) and a high concentrate diet (65% concentrate; High). **B** Principal component plot of ruminal fluid metabolome comparing cows without and with abomasal starch infusion to induce hindgut acidosis (HGA)

The distribution of samples on principal component plots based on the metabolic profiles is illustrated in Fig. 1. The high concentrate diet clustered separately from the Control diet; this observation was further confirmed by the statistical PERMANOVA result (*P* < 0.01). Additionally, as expected, the induction of HGA through infusion of starch did not have a significant impact on the metabolomic profile of the ruminal fluid according to the distribution of samples, and this was also demonstrated by the PERMANOVA analysis (*P* = 0.70). In general terms, these observations indicate that only the diet is expected to show specific impacts on the concentration of ruminal metabolites.

**Fig. 2:**
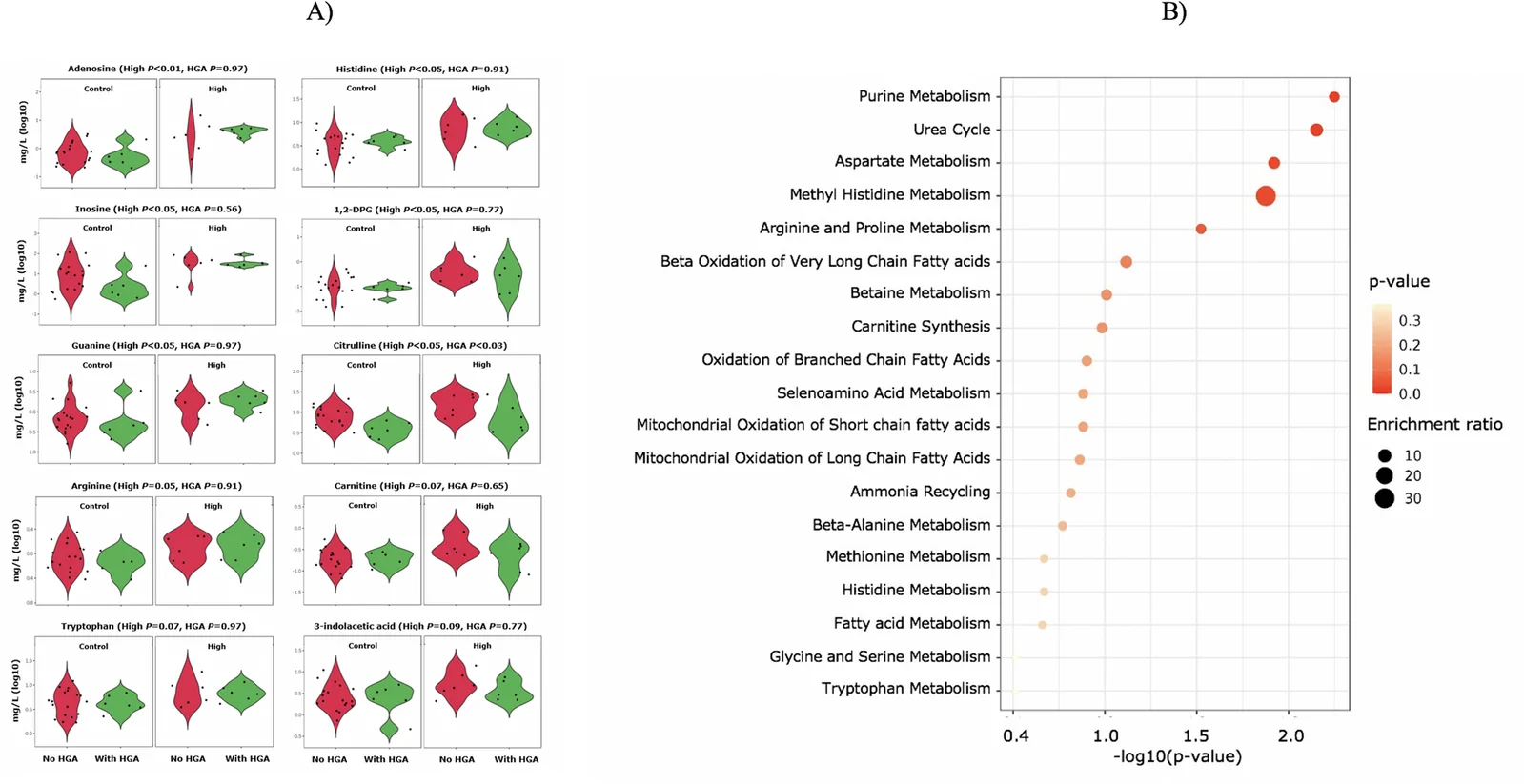
**A** Top ruminal metabolites affected by feeding a high concentrate diet (65% concentrate; High) or by abomasal starch infusion to induce hindgut acidosis (HGA) in Holsteins cows; the concentrations correspond to log converted and normalized values (mg/L) of the respective metabolite, DPG= dilinoleoyl-3-palmitoylglycerol; **B** Ruminal metabolic pathways predicted to be enriched based on the greater concentration of metabolites with the High diet

More specifically, ANOVA revealed that the change from the Control to High diet increased (*P* < 0.05) several metabolites, including adenosine, histidine, inosine, 1,2-dilinoleoeyl-3-palmitoylglycerol, guanine, citrulline and arginine. With this dietary change, tendencies (*P* = 0.07) for increased concentrations were observed for carnitine, tryptophan and 3-indoleacetic acid. The induction of HGA in cows only decreased (*P* < 0.05) the concentration of 2 metabolites in the rumen, namely trans-ferulic acid and citrulline. No interaction effect was found between high concentrate feeding and abomasal starch infusion on the ruminal metabolites (Fig. 2A). The increased concentration of metabolites due to the change from Control to High diet resulted in enrichment of several metabolic pathways (*P* < 0.05), primarily purine metabolism, urea cycle, aspartate metabolism, methyl histidine metabolism, arginine and proline metabolism. To a lower extent, enriched metabolic pathways included beta oxidation of long chain fatty acids, betaine metabolism and carnitine synthesis (Fig. 2B).

The rest of ruminal metabolites including those not affected by treatment are listed in the Supplementary material Table 1. Additionally, Supplementary material Fig. 3, shows the correlations between high concentrate feeding and several metabolites (*P* < 0.05) including adenosine (*r* = 0.676), histidine (*r* = 0.572), 1,2-dilinoleoeyl-3-palmitoylglycerol (*r* = 0.502), guanine (*r* = 0.493) and inosine (*r* = 0.492); whereas abomasal starch infusion correlated (*P* < 0.05) with trans-ferulic acid (*r* = − 0.59) and with 3-indolpropionic acid (*r* = − 0.42).

### Effects of high concentrate diet and abomasal starch infusion on the fecal metabolome

**Fig. 3:**
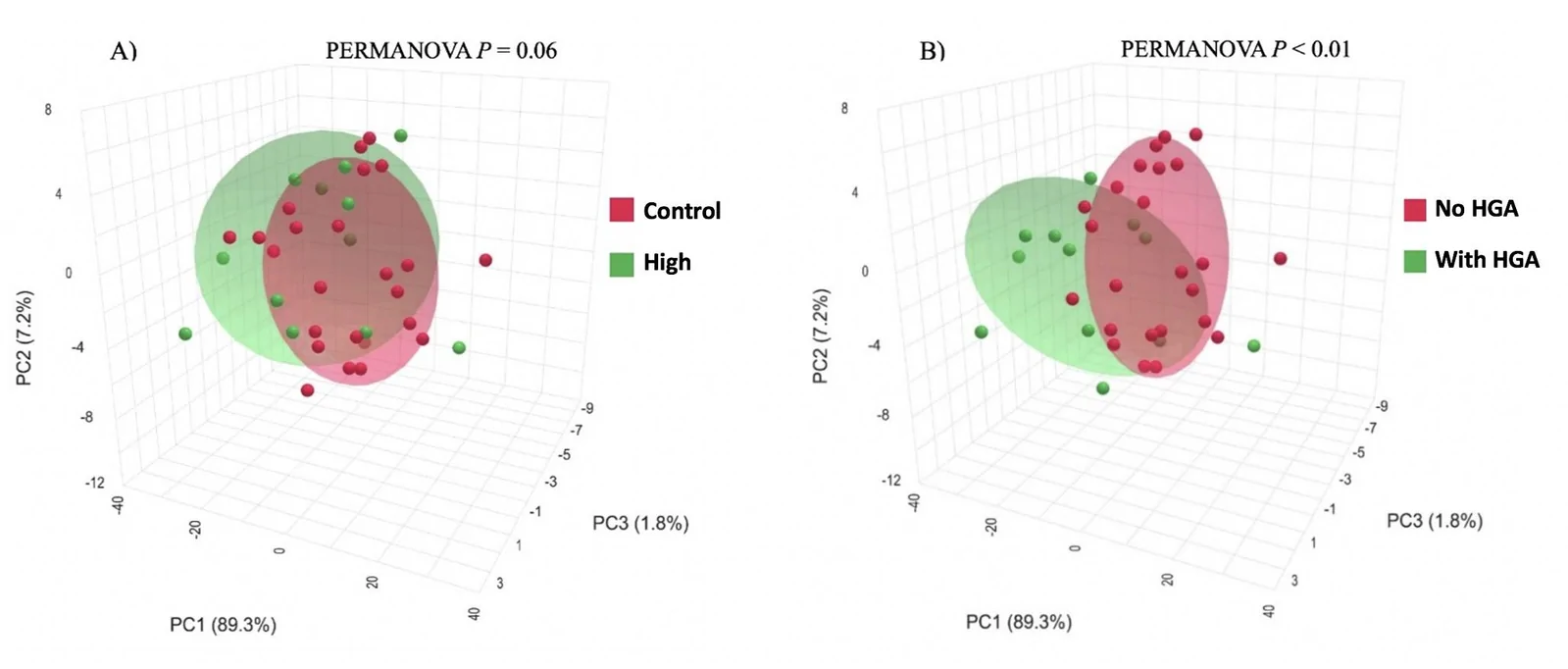
**A** Principal component plot of fecal metabolome comparing cows consuming a control diet with 40% concentrate (Control) and a high concentrate diet (65% concentrate; High). **B** Principal component plot of the fecal metabolome comparing cows without and with abomasal starch infusion to induce hindgut acidosis (HGA)

The distribution of fecal samples on principal component plots based on the metabolomic profiles is illustrated in Fig. 3. The High diet tended to cluster separately from the Control diet as also demonstrated by the PERMANOVA analysis (*P* = 0.06). Additionally, this analysis revealed that the induction of HGA had a significant impact on the metabolomic profile of the feces according to the distribution of samples, as also shown by the PERMANOVA results (*P* < 0.01). In general terms, these distributions of samples indicate that the level of concentrate as well as abomasal starch infusion are both expected to show impacts on the concentration of fecal metabolites, but with a greater effect of abomasal starch infusion.

**Fig. 4:**
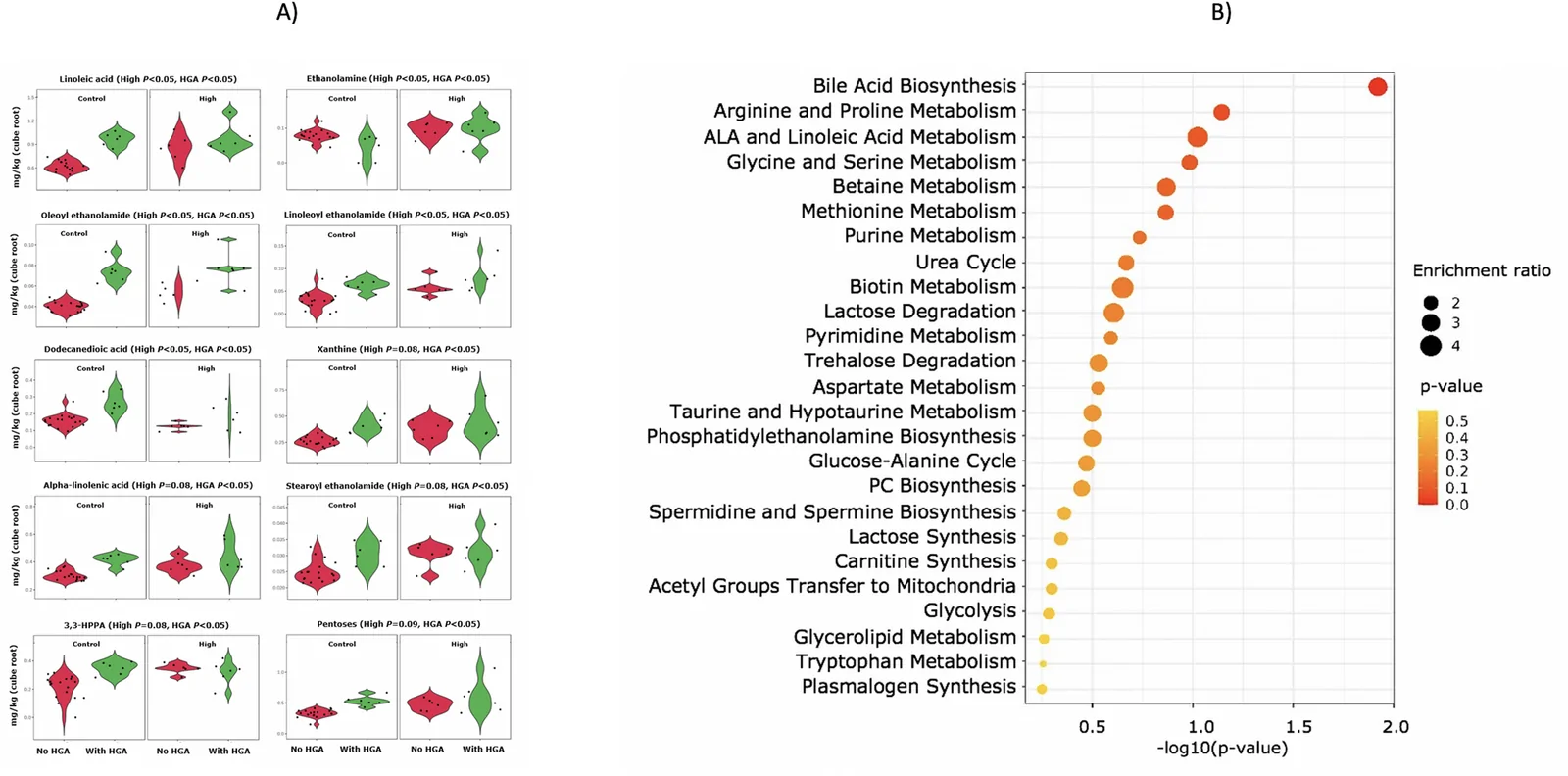
**A** Top fecal metabolites affected by feeding a high concentrate diet (65% concentrate; High) or by abomasal starch infusion to induce hindgut acidosis (HGA) in Holsteins cows; the concentrations correspond to cubic root converted and normalized values (mg/kg) of the respective metabolite, HPPA= hydroxyphenyl propionic acid; **B** Fecal metabolic pathways predicted to be enriched in cows based on the greater concentration of metabolites with HGA. ALA = alpha-linolenic acid

More specifically, the ANOVA results confirmed that High diet affected 10 metabolites. Namely, High increased (*P* < 0.05) the concentration of linoleic acid, ethanolamine, oleoyl ethanolamide, linoleoyl ethanolamine, dodecanedioic acid; and tended to increase (*P* < 0.10) xanthine, alpha-linolenic acid, stearoyl ethanolamide, 3,3-hydroxyphenyl propionic acid, and pentoses. This analysis also showed that the HGA affected 48 fecal metabolites, including an increase (*P* < 0.05) in the concentration of linoleic acid, ethanolamine, oleoyl ethanolamide, linoleoyl ethanolamide, xanthine, alpha-linoleic acid, stearoyl ethanolamide, 3,3-hydroxyphenyl propionic acid, pentoses, and reduced (*P* < 0.05) the concentration of dodecanedioic acid. No interaction was found between high concentrate feeding and abomasal starch infusion on fecal metabolites (Fig. 4A). The greater concentration of metabolites due to high concentrate feeding and abomasal starch infusion resulted in the enrichment (*P* < 0.05) of several metabolic pathways, including bile acid biosynthesis, arginine and proline metabolism, alpha-linoleic acid and linoleic acid metabolism, glycine and serine metabolism, betaine metabolism, methionine metabolism, purine metabolism, urea cycle, and biotin metabolism (Fig. 4B).

The complete list of detected fecal compounds is given in Supplementary material Table 2. Additionally, Supplementary material Fig. 4 shows the correlation between the high concentrate diet (*P* < 0.05) and several metabolites including linoleoyl ethanolamide (*r* = 0.527), linoleic acid (*r* = 0.507), xanthine (*r* = 0.480), alpha linolenic acid (*r* = 0.466), oleoyl ethanolamide (*r* = 0.463), pentoses (*r* = 0.456), stearoyl ethanolamide (*r* = 0.456), stachydrine (*r* = 0.436), 3,3-phenylpropionic acid (*r* = 0.435), and ethanolamine (*r* = 0.428). Whereas abomasal starch infusion correlated (*P* < 0.05) with several metabolites including oleoyl ethanolamide (*r* = 0.833), glycocholic acid (*r* = 0.783), hexoses (*r* = 0.774), cis-docosahexaenoic acid (*r* = 0.774), cholic acid (*r* = 0.773), stearic acid (*r* = 0.775), taurocholic acid (*r* = 0.731), creatine (*r* = 0.721), linoleic acid (*r* = 0.715), 3-indolepropionic acid (*r* = 0.708), uridine (*r* = 0.703), and N-stearoyl-D-sphingosine (*r* = 0.698).

### Effect of high concentrate diet and abomasal starch infusion on the blood serum metabolome

**Fig. 5:**
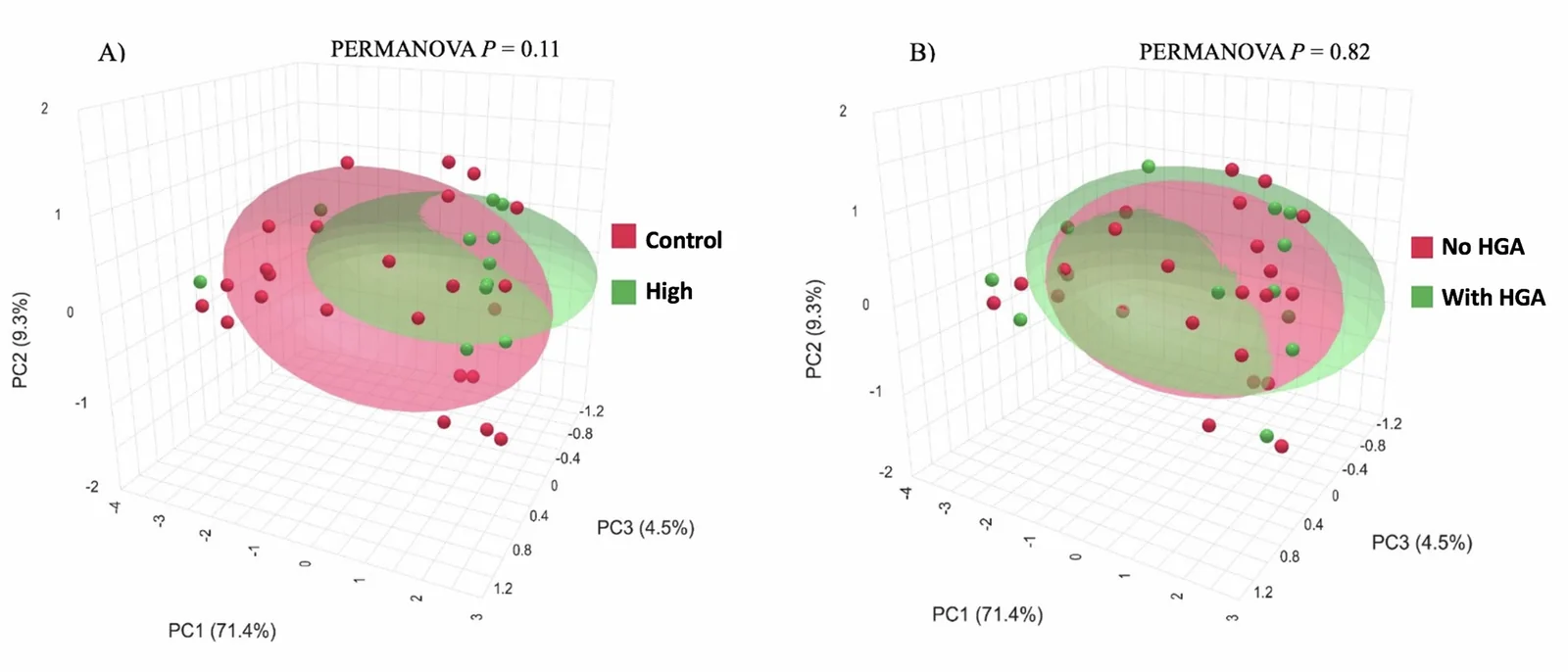
**A** Principal component plot of blood serum metabolome comparing cows consuming a control diet with 40% concentrate (Control) and a high concentrate diet (65%, High). **B** Principal component plot of blood serum metabolome comparing cows without and with abomasal starch infusion to induce hindgut acidosis (HGA)

The distribution of blood serum samples on principal component plots based on the metabolic profiles is illustrated in Fig. 5 illustrates. The High diet did not cluster separately from the Control diet; this observation agrees with results from PERMANOVA analysis (*P* = 0.11). Additionally, this analysis revealed that the induction of HGA did not have a significant impact on the metabolomic profile of the blood, as also demonstrated by PERMANOVA result (*P* = 0.80). Despite the lack of major profile shifts in this metabolome, detailed analysis of metabolite concentrations revealed important effects on several variables.

**Fig. 6:**
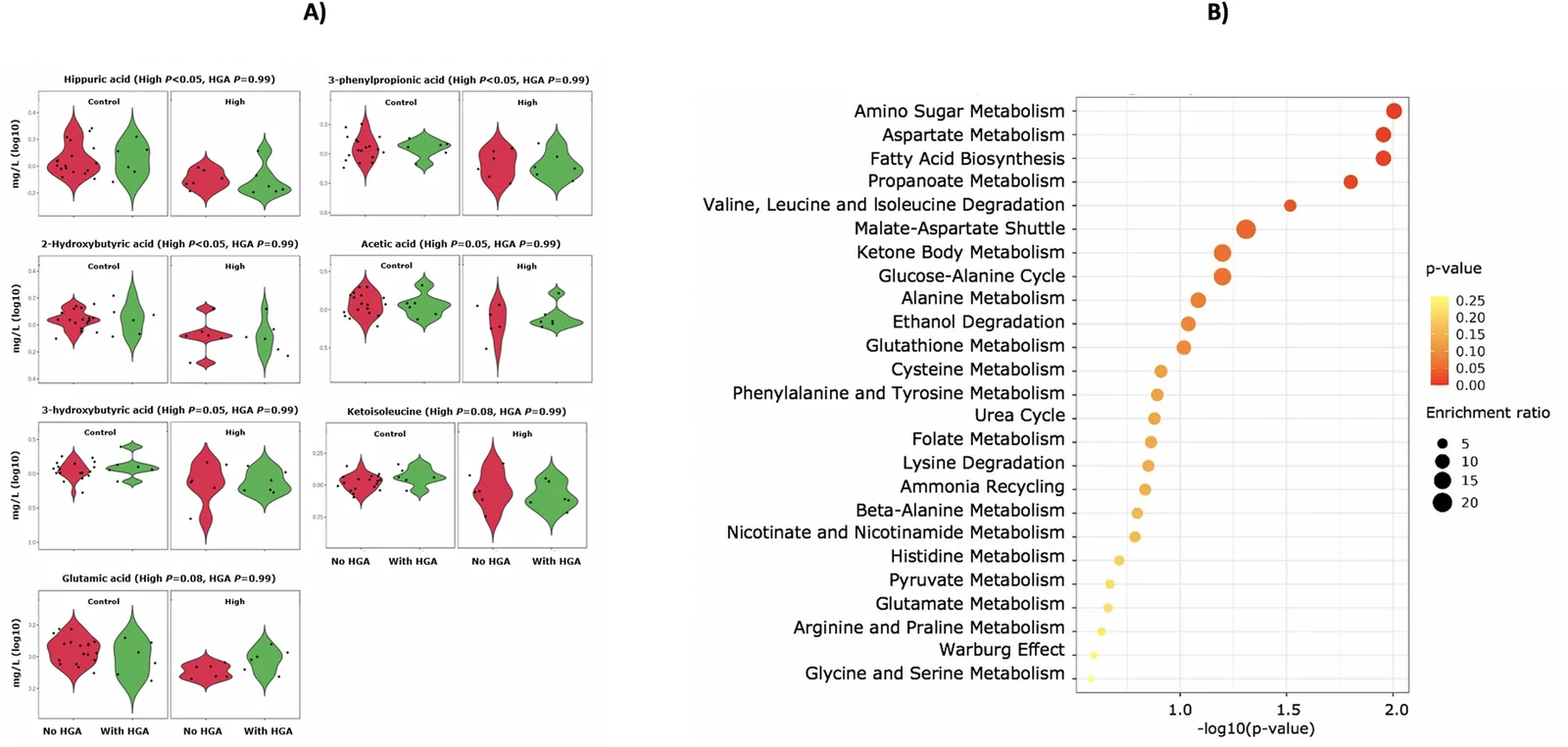
**A** Top blood serum metabolites affected by feeding a high concentrate diet (65% concentrate; High) or by abomasal starch infusion to induce hindgut acidosis (HGA) in Holsteins cows; the abundance corresponds to log converted and normalized concentration values (mg/L) of the respective metabolite; **B** Blood serum metabolic pathways predicted to be enriched based on the greater concentration of metabolites with the Control diet

Specifically, ANOVA revealed that when cows consumed the high concentrate diet, there were lower blood concentrations (*P* < 0.05) of hippuric acid, 3-phenylpropionic acid, 2-hydroxybutyric acid, acetic acid, and 3-hydroxybutyric acid; additionally, feeding the high concentrate diet tended to show lower (*P* < 0.05) concentrations of keto-isoleucine, and glutamic acid. However, abomasal starch infusion did not affect the concentration of the blood metabolites. Additionally, no interaction effect was found between high concentrate feeding and abomasal starch infusion on this metabolome (Fig. 6A). The greater concentration of blood metabolites with the Control diet compared to the High diet resulted in the enrichment (*P* < 0.05) of several metabolic pathways including amino sugar metabolism, aspartate metabolism, fatty acid biosynthesis, propanoate metabolism, valine, leucine and isoleucine degradation, and the malate-aspartate shuttle. Furthermore, enrichment of ketone body metabolism, glucose alanine cycle and alanine metabolism were found with the Control diet, although these latter pathways were enriched to a lower extent (Fig. 6B).

The rest of metabolites including those not affected by treatment are listed in Supplementary material Table 3. Additionally, Supplementary material Fig. 5 shows the correlation between high concentrate feeding (*P* < 0.05) and several blood metabolites such as hippuric acid (*r* = − 0.565), 3-phenylpropionic acid (*r* = − 0.558), 2-hydroxybutyric acid (*r* = − 0.534), acetic acid (*r* = − 0.496), and 3-hydroxybutyric acid (*r* = − 0.470). The abomasal starch infusion showed low correlation with blood metabolites (*r* < 0.400).

## Discussion

Despite extensive research evaluating the impacts of high concentrate feeding (Plaizier et al., 2018), there is considerably less information related to the effects of increased hindgut starch supply in ruminants (Abeyta et al. 2023b; Linder et al., 2025; Biber et al., 2026). Our results contribute to understanding not only the separate impacts but also the effects of both conditions. The preliminary findings reported in the companion paper (Biber et al., 2026) demonstrated that high concentrate feeding and the increased hindgut starch supply had separate and interactive impacts on foregut and hindgut fermentation profile as well as on the gut microbial communities. The High diet also shifted the volatile fatty acid profile of the rumen and hindgut. Therefore, this study was designed to evaluate if such preliminary findings regarding such impacts may also affect the metabolome as well as the underlying metabolic pathways of the ruminal fluid, the feces, and the blood of the cows.

According to our hypothesis, the high concentrate diet had a direct impact on the ruminal metabolome. This shift is expected, given the greater feed intake and the increased intake of rapidly fermentable carbohydrates in cows consuming the High diet (about 2,300 g/d of additional starch). The greater availability of starch enhanced the energy supply favouring microbial growth and metabolism increasing the proportion of ruminal propionate (Biber et al., 2026), but this also leads to low ruminal pH changing the rumen milieu. The drop of ruminal pH leads to greater microbial death and lysis as well as turnover of microbes and rumen epithelium. These impacts shifted the microbiome and related metabolites, thus resulting in enrichment of several metabolic pathways including purine metabolism, urea cycle, aspartate metabolism, methyl histidine metabolism, arginine and proline metabolism and beta oxidation of fatty acids.

Methyl histidine metabolism, the ruminal metabolic pathway with greatest enrichment ratio found in cows consuming the High diet, likely reflects intense microbial protein turnover due to changes in ruminal pH. Methyl histidine is also associated with dietary histidine metabolism, a crucial amino acid in dairy cattle (Schwab & Broderick, 2017). Thus, reflecting the greater feed and protein intake in cows fed the High diet (around 700 g more protein per day, and additional 3.35 kg/day of feed) compared to the Control diet. The enrichment of methyl histidine metabolism agrees with the correlation found between high concentrate feeding and several amino acids such as histidine, arginine and tryptophan. In addition, our results agree with Golder et al. (2012), who fed histidine in an acidosis challenge study and observed that the grain and histidine group produced higher ammonia concentration.

The increased microbial activity and microbial turnover agree with the enrichment of the metabolic pathway for purine metabolism. Purines are vital components of nucleic acids, which are extensively metabolized in the rumen (Reynal et al., 2005). Additionally, the increased supply of dietary protein and carbohydrates likely enhanced microbial growth and turnover, which increased the metabolism of purines (Broderick & Merchen, 1992). Thus, with the High diet, the increased supply of energy and protein may have contributed to greater microbial protein synthesis and metabolism of purines in rumen. This finding is in line with the positive correlations found between the High diet and several nucleotides, such as adenosine, guanine and uridine.

Another product of rumen microbial metabolism is ammonia (Hristov & Ropp, 2003). Part of the ammonia is recycled through the liver, then it is converted to urea (Lapierre & Lobley, 2001; Reynolds & Kristensen, 2008), which is transported to the kidneys, where it is either excreted or recycled back to the rumen (Reynolds, 1992). Although ruminal ammonia was not measured in this study, our findings show enrichment of the metabolic pathway of urea cycle in cows consuming the high concentrate diet. Additionally, there was enrichment of other pathways associated to ruminal ammonia generation in cows fed the high concentrate diet. For example, the metabolism of arginine into citrulline (increased in both high concentrate feeding and abomasal starch infusion) is performed by bacteria under low pH because it generates ammonia, helping raise the pH to favour conditions needed for bacterial survival (Vrancken et al., 2009).

From a systemic health perspective, our findings regarding shifts in ruminal metabolic pathways due to high concentrate feeding suggest potential long-term negative impacts. For example, the extensive microbial metabolism and increased urea cycle may represent greater workload for the liver and kidneys, which may lead to metabolic stress. Moreover, if ammonia is not efficiently detoxified, it may lead to chronic subclinical ammonia overload, increasing the risk of systemic health complications including impairment of normal activity of the nervous system, hormonal activity, reproduction function, or influence blood pH (Visek, 1984).

In the fecal metabolome, as hypothesized, concomitant effects of high concentrate feeding and abomasal starch infusion were observed, although the effects of high concentrate feeding were relatively milder compared to the impacts of abomasal starch infusion. These impacts agree with the observed shifts in the volatile fatty acids and lactate profile due to abomasal starch infusion as well as the change in the fecal microbiome (Biber et al., 2026). Specifically, abomasal starch infusion alone increased the concentration of 38 metabolites, resulting in enrichment of several metabolic pathways. For example, bile acids biosynthesis is an important metabolic pathway not only because of the role of bile in fat metabolism (Pacífico et al., 2021); but also because bile serve as crucial signalling molecules (Thomas et al., 2008; Hou et al., 2023). Research has shown the role of bile acids beyond their traditional function in fat absorption and cholesterol homeostasis (Sarkar et al., 2016). For example, bile acids activate various receptors, including the G-protein-coupled bile acid receptor 5 in the liver and peripheral tissues (Hou et al., 2023). This is reflected in the modulation of glucose and lipid metabolism and maintaining energy homeostasis (Wu et al., 2021; Ahmad & Haeusler, 2019). Therefore, enrichment in the synthesis of bile acids may be an effect of the increased starch overload following abomasal infusions, activating energy homeostatic mechanisms to metabolize glucose. However, it is important to note that chronically elevated levels of bile acids in the cows may increase the risk of systemic health issues in the long term. For example, high levels of bile acids have been shown to trigger specific signalling pathways that disrupt barrier function and increase intestinal permeability (Calzadilla et al., 2022). Additionally, research has shown that certain gut bacterial phyla such as Firmicutes, Fusobacteria, Actinobacteria, and Bacteroidetes exhibit significant inhibition in the presence of excessive bile acids (Peng et al., 2024).

Biotin metabolism, another metabolic pathway with high enrichment ratio found in the present experiment, is an important process in the lactating cow and microbial metabolism. For example, biotin is involved in several processes including carbohydrate metabolism. Specifically, pyruvate, the end product of glycolysis, is converted to oxaloacetate, whereby biotin serves as cofactor for the enzyme pyruvate carboxylase (Rodríguez-Fuentes et al., 2007). Additionally, in *de novo* fatty acid biosynthesis, acetyl CoA is converted to malonyl CoA, where biotin acts as a cofactor for the enzyme acetyl CoA carboxylase (Lee et al., 2008). Malonyl-CoA is a crucial molecule for the synthesis of fatty acids, including branched-chain fatty acids produced by gut bacteria.

Several metabolites increased due to acidotic condition either in the rumen or hindgut. Among these metabolites, fecal ethanolamine increased in high concentrate feeding and abomasal starch infusion. The increased ethanolamine agrees with the greater levels of fecal lactate and shifts in the microbiome (Biber et al., 2026), which are common signs of gut dysbiosis (Blake et al., 2019). Thus, this metabolite may be considered as a potential biomarker in cows suffering from low pH in both the rumen and hindgut. Greater ethanolamine may increase the risk for systemic health complications in the long term. The accumulation of ethanolamine in the gut may pose damage to gut permeability, increase inflammation, and lead to dysfunction in glucose metabolism (Fang et al. 2023). Thus, chronic accumulation of this biogenic amine may hamper cattle systemic health and production performance. Fang et al. (2023) also showed that reversing the complications caused by accumulation of ethanolamine can only be achieved when restoring to low levels. Other researchers have reported an increase in ethanolamine when cows consume increased levels of barley grain (Saleem et al., 2012). The latter authors also reported that ethanolamine is derived from phosphatidylethanolamine, which is abundant in membranes of shed enterocytes (Koichi et al., 1974). In addition, ethanolamine can be used by pathogenic bacteria (enterohemorrhagic *Escherichia coli* strain O157:H7) as a nitrogen source, conferring growth advantage over commensal microbiota (Saleem et al., 2012). For example, reports show that proliferation of the pathogens *Salmonella enterica* and *Escherichia coli* is promoted by ethanolamine (Bertin et al., 2011; Thiennimitr et al., 2011).

In the blood metabolome, our observations revealed enrichment of metabolic pathways for amino sugar metabolism, amino acid metabolism, fatty acid biosynthesis, propanoate metabolism, malate-aspartate shuttle and ketone body metabolism in cows consuming the Control diet. The metabolic pathway for fatty acid synthesis is essential in dairy cows, contributing to conversion of ruminal acetate into fatty acids (Smith et al., 2018). This metabolic process occurs primarily in the liver and adipose tissue. Then, *de novo* synthesized fatty acids are transported into the bloodstream for energy storage or cellular utilization (Liu et al., 2014). In dairy cows, milk is one of the most relevant fates of synthesized fat (Glascock & Welch, 1974). Thus, with the Control diet containing greater forage, the greater supply of ruminal acetate compared to High that we reported in the companion report (Biber et al., 2026), may have stimulated enrichment of this metabolic pathway. The greater supply of acetate in cows consuming the Control diet was also confirmed by the correlation analysis, where blood acetate negatively correlated with high concentrate feeding, indicating that the High diet led to lowered blood acetate. Additionally, adequate supply of protein when consuming the Control diet may explain the enrichment of metabolic pathways for the metabolism of absorbed amino acids (Lapierre et al., 2012).

Another metabolic pathway enhanced with the Control diet was the malate-aspartate shuttle, a mechanism for utilization of cytosolic glycolytic NADH (Holeček, 2023). For the cow, it is a crucial pathway because the NADH produced in the cytosol needs to be integrated into the electron transport chain in an efficient manner. In fact, this pathway is more efficient compared to the alternative mechanism glycerol 3-phosphate shuttle (McKenna et al., 2006). The enhancement in this metabolic pathway may be a result of lower NADH production with the Control diet compared to the High diet. Consequently, cows fed the Control diet have to use more efficient mechanisms for the utilization of the high-energy electrons from cytosolic NADH (Eder et al., 2020). The blood metabolic pathway differences observed due to diet may also have implications in the long term. For example, the greater efficiency in energy utilization with the Control diet compared to the High diet suggests that high concentrate diets may lead to lower efficiency in cellular use of dietary energy, with potential impacts on feed efficiency.

Contrasting our hypothesis, abomasal starch infusion did not shift the blood metabolome. This might be because the hindgut fermentation products are not completely absorbed, and are partly excreted, whereas ruminal fermentation end-products are extensively absorbed and directly transported into portal circulation (Sanz-Fernandez et al., 2024). Additionally, the reduction of hindgut pH due to abomasal starch infusion was not as low as in the rumen, thus the metabolic shift in the hindgut may have not been severe enough to influence the blood metabolome.

It is worth noting that some potential limitations of the study may include the relatively short washout period; a longer washout period could contribute to lower the risk of possible carry-over effects. The lack of milk data with sufficient statistical power as well as the lack of ruminal ammonia measurements may also be considered as potential limitations of the present study. Readers should also take into account that the change in the concentrate levels between the two diets also resulted in changes in other nutrients (besides starch) and potentially specific changes in amino acid or fatty acid profiles between the rations.

## Conclusion

High concentrate feeding affected the ruminal, fecal and blood metabolomes. In the rumen, the high concentrate diet enhanced the metabolic pathways for purine metabolism, urea cycle, methyl histidine metabolism, amino acid metabolism and beta oxidation of long chain fatty acids. In feces, both high concentrate diet and abomasal starch infusion had impacts, but abomasal starch infusion influenced the fecal metabolome to a greater extent, enhancing metabolic pathways for bile acid biosynthesis, amino acid metabolism, fatty acid metabolism, purine metabolism, urea cycle and biotin metabolism. The increased fecal ethanolamine may be considered as a biomarker in cows having low pH in both the rumen and hindgut. In the blood, the effect of high concentrate feeding was more evident compared to the effects of abomasal starch infusion, with enriched metabolic pathways for amino sugar metabolism, fatty acid biosynthesis, and amino acid metabolism; in particular, the enrichment of the malate-aspartate shuttle for the Control diet suggests enhancement of more efficient mechanisms for the use of cytosolic NADH when cows consume the Control diet. Overall, results contribute to understanding the effects of high concentrate feeding and increased starch bypassing ruminal degradation, emphasizing the need of maintaining adequate gut health in cows to prevent metabolomic alterations.

## Supplementary Information

Below is the link to the electronic supplementary material.

Supplementary material 1 (DOCX 14.3 kb)

## Data Availability

Complete metabolome data of this paper are available via https://doi.org/10.17632/2w4fk6vc3k.2.
