# Supplementary material for: Metabolome analyses of the rumen, feces and blood reveal separate and simultaneous impacts of increased dietary concentrate and hindgut starch flow in Holstein cows
Source: Metabolomics. 2026 Oct 3;22(5):163. doi: 10.1007/s11306-026-02519-0 (PMC13633985; doi:10.1007/s11306-026-02519-0)
Supplement: Supplementary file 1 [file 11306_2026_2519_MOESM1_ESM.docx]

**Supplementary material** can be found at https://doi.org/10.17632/3m9p7s9wk3.3

**Supplementary Material Captions**

**Supplementary Fig. 1**. Experimental outline showing the assignment of treatments within each of the 2 experimental periods. The Control diet contained 40% concentrate. Ruminal acidification was induced with a high concentrate diet (65% concentrate; High). Hindgut acidosis (HGA) was induced through daily abomasal infusion of starch. Each period consisted of 3 weeks. Between the 2 experimental periods, there was a washout interval of 21 days.

**Supplementary Fig. 2** Ruminal pH (duration of pH<5.8; A) and fecal pH (B) in cows fed a normal lactation diet containing 40% concentrate (Control) or a high concentrate diet (65% concentrate; High) with or without abomasal infusion of 3 kg of starch to induce hindgut acidosis (HGA).

**Supplementary Fig. 3** A) Correlation of ruminal metabolites with high concentrate feeding (65% concentrate; High); B) Correlation of ruminal metabolites with hindgut acidosis (HGA); positive correlations indicate an increase in concentration with the acidotic condition.

**Supplementary Fig. 4** A) Correlation of fecal metabolites with high concentrate feeding (65% concentrate; High); B) Correlation of fecal metabolites with hindgut acidosis (HGA); positive correlations indicate an increase in concentration with the acidotic condition.

**Supplementary Fig. 5** A) Correlation of blood serum metabolites with high concentrate feeding (65% concentrate; High); B) Correlation of blood serum metabolites with hindgut acidosis (HGA); positive correlations indicate an increase in concentration with the acidotic condition.

**Supplementary Table 1.** Effect of high concentrate feeding (65% concentrate; High) and hindgut acidosis (HGA) on ruminal metabolites in Holstein cows; results include the raw P-values as well as the Benjamini-Hochberg adjusted P-values.

**Supplementary Table 2.** Effect of high concentrate feeding (65% concentrate; High) and hindgut acidosis (HGA) on fecal metabolites in Holstein cows; results include the raw P-values as well as the Benjamini-Hochberg adjusted P-values.

**Supplementary Table 3.** Effect of high concentrate feeding (65% concentrate; High) and hindgut acidosis (HGA) on blood serum metabolites in Holstein cows; tesults include the raw P-values as well as the Benjamini-Hochberg adjusted P-values.
